# A Descriptive Survey Investigating the Impact of the COVID-19 Pandemic on the Public’s Perception of Healthcare Professionals

**DOI:** 10.7759/cureus.41703

**Published:** 2023-07-11

**Authors:** Laurence Stolzenberg, Austin Huang, Mohammad Usman, Gordon MacGregor

**Affiliations:** 1 Orthopedic Surgery, Alabama College of Osteopathic Medicine, Dothan, USA; 2 Neurology, Alabama College of Osteopathic Medicine, Dothan, USA; 3 Anesthesiology, Alabama College of Osteopathic Medicine, Dothan, USA; 4 Pharmacology, Alabama College of Osteopathic Medicine, Dothan, USA

**Keywords:** epidemiology, covid-19, health policy, infectious disease, public health

## Abstract

The COVID-19 pandemic brought immense attention to the healthcare system and its workers. While much research has been completed about the effects of COVID-19 on the healthcare system, little exists about how the opinions of patients have been altered by this pandemic. We decided to further investigate how the public opinion of healthcare workers (HCWs) has changed to better understand how best we can serve society.

The key takeaway from the data was that both the levels of perceived trustworthiness and respectability of healthcare workers decreased following the pandemic. Data showed that the level of perceived respectability decreased from an average of 7.84 to 7.30 and the level of perceived trustworthiness from 7.38 to 6.54, all of these values out of 10. While these changes were not enormous, they demonstrate a striking trend and were found to be significant through a paired t-test. Finally, respondents were also queried about their level of desire in pursuing healthcare as a career field and overwhelmingly there was little interest, with an average level of 1.24 out of 10.

We believe our data and results show important trends that all HCWs should be aware of; notably decreasing interest in the field, reduced trust, and decrease in respect, all of which will require further study and analysis. We must consider the current environment in which small mistakes or mistrust can have grave consequences on public health and patient compliance. In addition, the lack of interest in joining the medical community is concerning considering the large efflux of workers leaving the profession. Future studies could focus on how to increase trust in HCWs or attract more people to the healthcare field.

## Introduction

Patient satisfaction and opinions are very important aspects of medicine that must always be considered. A patient’s lack of, or low level of, satisfaction directly impacts their outcomes as their compliance and likelihood of seeking medical attention may be negatively affected. Consequently, research on patient experiences, satisfaction, and perspectives have gained increasing attention including the first national, standardized, publicly reported survey of patients’ perspectives of hospital care, called The Hospital Consumer Assessment of Healthcare Providers and Systems (HCAHPS) survey [1]. Since the development and implementation of this survey, multiple research projects have utilized this data in order to improve their quality of care and how patients perceive their care [2,3]. With these calls to make healthcare more patient-centered, patient perception has become a key performance metric and is even utilized to determine hospitals’ compensation. A common theme that has been emphasized in the literature is the focus on the importance of effective communication between healthcare providers and patients [2,4,5]. This increased focus on patient-centered care has resulted in a shift in provider practices, allowing patients to have a more active role in their care, leading to changes in how facilities are run and reimbursed all with the goal of increasing patient outcomes [6].

The coronavirus disease 2019 (COVID-19) pandemic drastically changed the medical field, with rapid changes and issues including financial challenges for healthcare facilities, postponement of elective surgeries and non-urgent care, further development of telehealth, workforce shortages, and altered training [7-10]. Since the beginning of the COVID-19 pandemic, a strong focus was aimed at the impact on healthcare workers (HCWs). Research aimed at uncovering the negative impact of the pandemic on HCWs found important information, such as increasing rates of burnout, depression, anxiety, and suicide [11-16]. Of course, patients were directly impacted by the pandemic due to care being postponed, difficulty in obtaining follow-up visits, difficulties reaching hospitals by ambulance due to overuse of resources, and increased wait times. Patients also faced difficulties obtaining healthcare that respected the standard of care for acute and chronic illnesses and changes to medical facility policies such as visitor limitations [7,15,17]. However, research focusing on patient perceptions, opinions, or satisfaction of care since the beginning of the COVID-19 pandemic has been severely lacking. Some research has measured patient perceptions when it comes to the use of telehealth services due to the impact of the pandemic. Other research has investigated the impact of the pandemic on specific patient populations, such as rheumatic patients’ perceptions [18]. Considering the unprecedented nature of the COVID-19 pandemic, we were shocked to observe the significant void in information about patient opinions about all the different changes affecting the healthcare landscape in the United States.

With the continued push to improve the quality of healthcare via a more patient-centered mindset and with the unprecedented and unknown impact of COVID-19 on this push, we aim to explore this topic further. We hope to shed light on the impact of the COVID-19 pandemic on patient perspectives and opinions of healthcare professionals.

## Materials and methods

In this descriptive study, we aimed to collect participant opinions of healthcare professionals before and after the COVID-19 pandemic via a 16-tem Qualtrics survey. Before the study, multiple physicians in one hospital and one clinic in Alabama, and two clinics in Florida were recruited to direct their patients to fill out the survey. These physicians were provided with recruitment flyers to post at their offices for patients to see or to directly provide to patients. Additionally, these flyers were posted with permission around the same healthcare facilities. A link to the survey was posted online via various social media platforms, including Reddit, Twitter, and Facebook. The survey was available for the public to fill out between May 2022 and October 2022.

The study population was anyone with access to the internet and who was over 21 years of age. We wanted a broad and generalized understanding of this topic and decided to impose as few inclusion criteria as possible. Our sample size was those who agreed to participate and complete our survey. Notably, there were 181 responses before the filtering process described in the results section. This sample size was not predetermined and was simply set by the number of people who agreed and completed our survey in the predetermined time. We had set the maximum possible number of respondents to 500, after discussion with our institutional review board, but never reached that number. If we had reached this maximum, we would have closed the survey early, although it was not a goal or expected number of responses.

The statistical analysis of the data collected throughout this survey was done using Microsoft Excel. Additionally, for the variation of perceived respectability and trustworthiness, the p-values were calculated using a paired t-test.

Our survey collected several variables. First, we collected some basic information including the time required for completion of our survey, the age of the respondent, and gender (male, female, prefer not to disclose, self-disclose with a text box). Next came our main questions about the perceived trustworthiness and respectability of healthcare workers, requesting their opinion before and after the pandemic, both using a 10 scale with 0 being no perceived respectability or trustworthiness and 10 being extreme perceived respectability and trustworthiness. We asked respondents if they were healthcare workers before the pandemic, and if they were healthcare workers now. We asked respondents if they would consider becoming healthcare workers in the future, using a scale from 0-10, where 0 meant absolutely not, and 10 meant actively planning to become one. We asked respondents if they had a family physician before the pandemic, and if they had one now. Finally, we asked respondents if they previously believed healthcare workers should be involved in politics before the pandemic, and what their opinion was on this topic now, again using a 10 scale with 0 being absolutely not, and 10 being a resounding yes.

Inclusion and exclusion criteria 

Those included were men and women, 21 years old and above. Anyone below that age was automatically excluded from the study, as was anyone who did not accept the informed consent question at the start of the survey. If it was determined that the participant did not fulfill the requirements, the survey automatically closed. The survey was completely anonymous and confidential. No identifying information was collected.

## Results

The survey received a total of 181 responses. After filtering out results that answered the entire survey in less than 60 seconds, that had not answered all questions, we were left with 122 (67.4%) responses.

The average age of respondents was 34.7 years old (range 21-71), with 39 males (32.0%), 75 females (61.5%), 1 intersex (<1%), and 7 who preferred not to disclose (5.7%).

Subjects were asked how trustworthy they believed medical professionals were and their level of respect for healthcare workers before the pandemic (Table 1). The perceived level of trustworthiness before the pandemic (on a scale of 1 to 10) was 7.38 ± 1.95 (n=122). The level of respect for healthcare workers before the pandemic was 7.84 ± 1.95 (n=122) (Table 1).

**Table 1:** Self-reported level of perceived trustworthiness of healthcare workers before and after the pandemic. This information was self-reported by participants using the numerical scale, where 0 meant no perceived trustworthiness at all, and 10 was extreme perceived trustworthiness.

Scores from 1-10	Number of responses before the pandemic	Number of responses after the pandemic
0	2	7
1	0	4
2	0	6
3	7	7
4	2	6
5	5	5
6	10	9
7	24	12
8	39	32
9	23	22
10	10	12
Total responses	122	122
Average	7.38 ± 1.95	6.54 ± 2.92

Subjects were asked their opinion on the same question at the present day, two years after the start of the pandemic (Table 2). The respondents' perceived trustworthiness and respect for healthcare workers both decreased when responding for the current day, two years after the beginning of the COVID-19 pandemic. The perceived level of trustworthiness of healthcare workers after the pandemic (on a scale of 1 to 10) was 6.54 ± 2.92 (n=122), a decrease of 0.85 (p<0.0001). The level of respect for healthcare workers during that same timeframe was 7.30 ± 2.83 (n=122), a decrease of 0.54 (p<0.05) respectively (Table 2). The p-values were calculated using a paired t-test.

**Table 2:** Self-reported level of perceived respectability of healthcare workers before and after the pandemic. This information was self-reported by participants using the numerical scale, where 0 meant no respectability at all, and 10 was extreme respectability.

Scores from 1-10	Number of responses before the pandemic	Number of responses after the pandemic
0	0	6
1	0	1
2	1	4
3	6	5
4	4	6
5	5	7
6	7	6
7	15	7
8	32	25
9	27	27
10	25	28
Total responses	122	122
Average	7.84 ± 1.95	7.30 ± 2.83

Another statistic that was collected was whether respondents were healthcare workers themselves. Before the pandemic, 107 of the 122 respondents denied being part of healthcare, and 15 endorsed being HCWs (12.3%). Following the pandemic, 14 endorsed being healthcare workers (11.5%). Further, of all respondents, 85 had personal physicians, meaning they were followed by a primary care physician, before the pandemic (69.7%) versus 81 after the pandemic (66.4%), representing a decrease of 3.3%.

Respondents were questioned on whether they would consider becoming a healthcare professional as a career choice now. The scale was set at 0 being “absolutely not” and 10 “planning to do so.” The average value picked by respondents who were not healthcare workers was 1.24 ± 1.95 (n=105).

The final statistics that were collected were about the public opinion on physicians participating in public policy and politics. Respondents were asked early in the survey on a scale of 0-10, if they believed physicians should participate in public policy. The average opinion of respondents was 6.42 ± 2.97 (n=122). When the same question was asked at the end of the survey about the current day, after the pandemic, respondents’ average opinion did not change (p = 0.917) and was 6.38 ± 3.24 (n=122).

## Discussion

The purpose of this study was to shed light on the impact of the COVID-19 pandemic on patient perspectives and opinions of healthcare professionals. Considering how vital the patient-physician relationship is when providing healthcare, it is critical that healthcare professionals understand the opinions and perspectives of those they are treating. Our results show that, as we will discuss below, our patients' opinions may have gone in a direction contrary to what would permit ideal care, which would come with a slew of issues we will discuss in the following sections.

**Figure 1:**
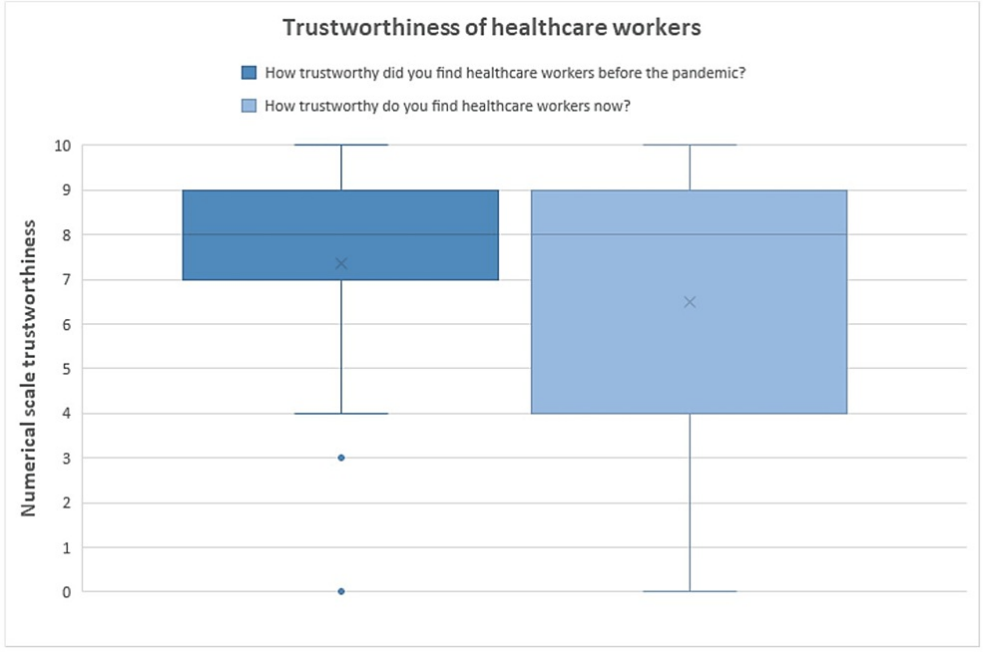
Trustworthiness of healthcare workers, before and after the pandemic. Box and whisker plot showing quartiles. Before the pandemic: Min: 0, Max: 10, Median: 8, Mean 7.38 ± 1.95, 95% confidence interval 7.38 ± 0.35. After the pandemic: Min: 0, Max: 10, Median: 8, Mean 6.54 ± 2.92, 95% confidence interval 6.54 ± 0.52. The interquartile range (IQR) is defined as the distance between the 1st quartile and the 3rd quartile. The X on the graph represents the Mean.

Regarding the public's perceived trustworthiness of healthcare workers before and after the pandemic, a marked decrease in these measures was observed (Figure 1). While our research showed a decrease, there was no significant change in the distribution of perceived trustworthiness. Not only is this significant for all clinicians and practitioners, as patient trust is a very important component in patient satisfaction, but is also strongly and intrinsically linked with compliance, both with public health directives and physician instructions [19]. We hypothesize that the divided response, and often contradictory instructions from public health agencies contributed to certain portions of society losing some measure of trust in healthcare professionals. There are a few clear examples of these contradictory instructions, such as the changing CDC directives early during the pandemic [20], as well as the initial contraindication to N95 mask use early in the pandemic in the United States and Canada [21], likely driven by worries of insufficient stock for clinicians, which was suddenly reversed with a strong publicity campaign about mask use.

**Figure 2:**
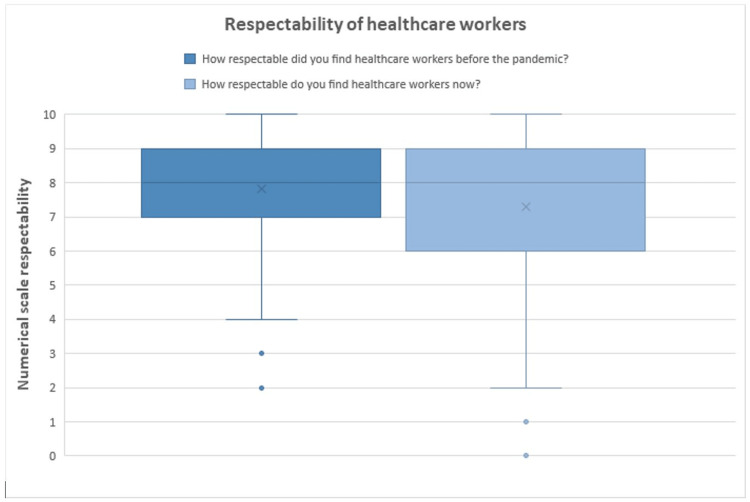
Perceived respectability of healthcare workers, before and after the pandemic. Box and whisker plot showing quartiles. Before the pandemic: Min: 2, Max: 10, Median: 8, Mean 7.84 ± 1.95, 95% confidence interval 7.84 ± 0.35. After the pandemic: Min: 0, Max: 10, Median: 8, Mean 7.30 ± 2.83, 95% confidence interval 7.30 ± 0.51. The interquartile range (IQR) is defined as the distance between the 1st quartile and the 3rd quartile. The X on the graph represents the Mean.

This same mechanism very well could have played a part in the loss of respect for healthcare workers, although other mechanisms could be suggested here, such as a disproportionate number of mortality and morbidity in ethnic and racial minorities in the United States caused by the pandemic [22]. This could obviously lead to marginalized populations losing trust and respect, as we observed in our results (Figure 2).

We collected statistics about whether respondents were, or not, healthcare workers both before and after the pandemic. Although our results show a slight decrease in the number of people working in this field after the pandemic, the variation was so minimal as to be considered insignificant. One interesting fact is that approximately 12% of our respondents were healthcare workers, which matched the national average [23]. This hinted that our sample was, for this point, representative of the population at large. Of note, the same trend was not present for a query about respondents who had a personal physician before the pandemic 69.7%, which then decreased by 3.3% to 66.4%. Although this is not a drastic drop, it may be linked to the significant number of healthcare workers leaving the field, and decreased number of people wishing to enter the field which will be discussed further in this paper, leading to physician shortages [24]. Another hypothesis is that some patients appear to have lost trust in the medical community and may have chosen to no longer seek regular primary care. There is some data to support this hypothesis, although nothing is extremely credible or concrete, so it remains a theory [25].

When reviewing whether respondents desired to start a career in healthcare, we were absolutely shocked by the extremely low desire to join this field, notably 1.24 ± 1.95 out of 10. It becomes clear that the average interest in this field is terribly low. There are innumerable possible causes for this, from dangerous working conditions, decreasing remuneration, immense student debt, the time investment required to begin, and very high levels of legal liability in the United States. Although the exact causes for this would be ground for numerous other papers, it becomes obvious that this can, and possibly shall lead to shortages of all healthcare workers and physicians in particular [24]. We would simply caution in keeping in mind the few limitations to our study discussed below.

Finally, we queried respondents about their opinion on whether healthcare workers should have a place in public policy. To our surprise, in contrast with most of the other questions in our survey, there was very little variation in opinion between before and after the pandemic, and respondents seem on average relatively comfortable with HCWs participating in politics.

Limitations 

Our study has limitations. The sample size is small and may not reflect the general population due to participants requiring internet access to complete our survey. This data was collected at a single point in time, thus participant perceptions prior to the COVID-19 pandemic that they had to recall may not accurately reflect their true experience.

One bias that should be mentioned is that most of our survey participants were young, with an average age of 34.7 years. This could be considered a bias, as our sample may be slightly skewed towards a younger average age than society at large. This may be partly explained by our use of social media, including Facebook and Reddit, to gather data. These social media platforms are well known to skew towards younger populations. Although we did have flyers handed out in medical settings, respondents required internet access, which may have restricted some potential respondents from answering our survey. Additionally, a portion of our recruitment was done in a healthcare setting, as described above, which could introduce further bias. With all these limitations in mind, we believe that this study nonetheless brings new and useful information to the discussion of healthcare workers' place in public health, politics, and society in general. 

## Conclusions

As the COVID-19 pandemic had a significant impact on the healthcare field and on society at large, it is only expected for there to be significant variations in public opinion with respect to the field and the people who practice in it. However, the results show a concerning trend. While in an ideal world, there would have been an increase in both respect for and trust in healthcare professionals following the numerous challenges faced during this period. In practice, respondents demonstrated the exact opposite. While the absolute numbers showed the shift was not particularly large, it is a development that healthcare practitioners and physicians should be mindful of. Being able to create a relationship with your patients fundamentally relies on a level of trust and respect that goes both ways between the healthcare worker and the patient. If that relationship starts to break down, there may be significant repercussions with respect to healthcare outcomes and patient compliance. A patient who does not trust that their physician has their best interests at heart is less likely to follow the suggestions and prescriptions of their doctor, with potentially catastrophic consequences.

Finally, one piece of datum that was also significant when considered in the overall outlook of the future of American healthcare is the nearly complete rejection of the desire to become a healthcare worker. The profession was severely strained, with some facilities and states having staffing issues pushing hospitals nearly to the breaking point during the pandemic. Many healthcare systems are still suffering a variety of issues from adequate staffing to supply chain shortages. More research is desperately needed as to how best we can attract the next generation of those who may choose healthcare as a profession.
